# King Edward's Hospital Fund for London

**Published:** 1909-01-16

**Authors:** 


### KING EDWARD'S HOSPITAL FUND FOR LONDON.

A MEETING of the General Council of King Edward's Hospital Fund for London was held on the 11th inst., at the offices of the Fund, 7 Walbrook, E.C., at which there were present : Mr. Hugh Colin Smith (in the chair), the Rev. T. Bowman Stephenson, D.D., the Governor of the Bank of England (Mr. W. Middleton Campbell), the Right Hon. Sir Savile Crossley, Bart., the Right Hon. Sir Joseph Dimsdale, Bart., the Right Hon. Sir Ernest Cassel, the Right Hon. C. Stuart-Wortley, K.C., M.P., Sir W. S. Church, Bart., Sir Horace Marshall, Sir W. J. Collins, M.P., Mr. J. Danvers Power, Dr. Edwin Freshfield, Mr. Frederick M. Fry, Mr. John G. Griffiths, Mr. Albert G. Sandeman. A communication was received signed by his Royal Highness the President, appointing for the year 1909 the members of the General Council, the Finance Committee, the Distribution Committee, the Convalescent Homes Committee, and the Executive Committee, and the officers—viz., Hon. Treasurer, Lord Rothschild; Hon. Secretaries, Sir Savile Crossley, Bart., and Mr. Frederick M. Fry; Hon. Solicitors, Messrs. Freshfields; Hon. Auditors, Messrs. Deloitte, Plender, Griffiths and Co.; Secretary, Mr. Maynard. The resolutions providing for the work of the Fund for 1909, which were approved at the meeting of the President and General Council held at Marlborough House on December 14, 1908, were formally adopted.

